# Dose escalation by image-guided intensity-modulated radiotherapy leads to an increase in pain relief for spinal metastases: a comparison study with a regimen of 30 Gy in 10 fractions

**DOI:** 10.18632/oncotarget.18979

**Published:** 2017-07-04

**Authors:** Jinlan He, Jianghong Xiao, Xingchen Peng, Baofeng Duan, Yan Li, Ping Ai, Min Yao, Nianyong Chen

**Affiliations:** ^1^ Department of Radiation Oncology, Cancer Center and State Key Laboratory of Biotherapy, West China Hospital, Sichuan University, Chengdu, Sichuan 610041, China; ^2^ Department of Radiation Oncology, Case Comprehensive Cancer Center, University Hospitals and Case Western Reserve University School of Medicine, Cleveland, OH 44106, United States

**Keywords:** dose escalation, image-guided radiotherapy, intensity-modulated radiation therapy, pain relief, spinal metastasis

## Abstract

**Purpose:**

Under the existing condition that the optimum radiotherapy regimen for spinal metastases is controversial, this study investigates the benefits of dose escalation by image-guided intensity-modulated radiotherapy (IG-IMRT) with 60–66 Gy in 20–30 fractions for spinal metastases.

**Results:**

In the dose-escalation group, each D50 of planning gross tumor volume (PGTV) was above 60 Gy and each Dmax of spinal cord planning organ at risk volume (PRV) was below 48 Gy. The median biological effective dose (BED) of Dmax of spinal cord was lower in the dose-escalation group compared with that in the 30-Gy group (69.70 Gy vs. 83.16 Gy, *p* < 0.001). After one month and three months of the radiotherapy, pain responses were better in the dose-escalation group than those in the 30-Gy group (*p* = 0.005 and *p* = 0.024), and the complete pain relief rates were respectively 73.69% and 34.29% (*p* = 0.006), 73.69% and 41.38% (*p* = 0.028) in two compared groups. In the dose-escalation group, there is a trend of a longer duration of pain relief, a longer overall survival and a lower incidence of acute radiation toxicities. No late radiation toxicities were observed in both groups.

**Materials and Methods:**

Dosimetric parameters and clinical outcomes, including pain response, duration of pain relief, radiation toxicities and overall survival, were compared among twenty-five metastatic spinal lesions irradiated with the dose-escalation regimen and among forty-four lesions treated with the 30-Gy regimen.

**Conclusions:**

Conventionally-fractionated IG-IMRT for spinal metastases could escalate dose to the vertebral lesions while sparing the spinal cord, achieving a better pain relief without increasing radiation complications.

## INTRODUCTION

Bone is the third most common metastatic site of cancer [1], and spine is the most common affected bone [2]. About one third of cancer patients were found to have spinal metastases on autopsy [3]. Pain [4], motor and sensory dysfunction [5], and spinal cord compression [6] are the main detriments of spinal metastases, which impair the patients’ quality of life significantly.

Radiotherapy (RT) is the major treatment modality for spinal metastases [7]. It is efficacious in relieving pain, alleviating spinal cord compression, and preventing neurologic decline and pathological fracture [6]. To date, however, there is no consensus on the standard radiotherapy scheme for spinal metastases. The regimens most widely employed are 8 Gy/1fraction (f), 30 Gy/10f, and 40 Gy/20f [7], with which similar pain relief [8, 9] and equal function improvement [10–13] could be achieved. However, 30 Gy/10f remains the most commonly used one [14, 15]. Although the overall pain relief rate can reach 60%–70% with the above three schemes, only 20%–30% patients will gain complete pain relief [8, 16] and the median duration of pain relief is only 3–6 months [8, 9, 16]. Improvements in diagnostic and therapeutic strategies have prolonged the survival of cancer patients and motivated their pursuit of a long duration of pain relief or the complete pain relief [17].

Local control rate of spinal metastases tends to increase with the escalation of radiation dose [10, 18, 19]. On the other hand, pain relapse is related to the failure of local tumor control. Therefore, for patients with a life expectancy of more than 3–6 months, escalating dose to spinal metastases is expected to achieve a higher pain relief rate, and a longer duration of pain relief. However, since spinal metastasis is often adjacent to or encircling the spinal cord, conventional radiotherapy could hardly escalate dose to spinal metastasis while sparing the spinal cord [20]. Intensity-modulated radiation therapy (IMRT) can improve conformality to tumor and meanwhile reduce doses to normal tissues [21]. Image-guided radiotherapy (IGRT) can offer precise delivery by monitoring and correcting the set-up errors [22]. The combination of IMRT and IGRT makes it possible to escalate dose to spinal metastases.

Our previous study has shown the feasibility of applying cone beam computer tomography (CBCT) guided IMRT for dose escalation beyond 60 Gy [23]. In this study, we further determined the benefits of dose escalation to spinal metastases in dosimetric parameters and clinical outcomes with a larger sample size. We also compared the results with those treated with 30 Gy/10f scheme, aiming to clarify the superiority of the dose-escalation regimen and to provide a basis for clinical decision-making.

## RESULTS

### Patient characteristics

Patient characteristics are shown in Table 1. There was no significant difference between the two groups. Up to 80% lesions were painful (55/69) before radiotherapy. There was no significant difference in the proportion of painful lesions (*p* = 0.564) or the overall distribution of pain severity (*p* = 0.154).

**Table 1:** Patient characteristics between two radiotherapy groups

	Dose-escalation group (*n* = 25) No. of lesions (%)	30-Gy group (*n* = 44) No. of lesions (%)	*P*
Age (years)			0.066
< 60	22 (88.00)	30 (68.18)	
≥ 60	3 (12.00)	14 (31.82)	
Gender			0.828
Male	16 (64.00)	27 (61.36)	
Female	9 (36.00)	17 (38.64)	
ECOG performance status			0.074
0	10 (40.00)	9 (20.45)	
1–2	14 (56.00)	31 (70.45)	
3–4	1 (4.00)	4 (9.09)	
Primary tumor ^a^			0.999
Unfavorable	22 (88.00)	40 (90.91)	
Favorable	3 (12.00)	4 (9.09)	
Spinal metastasis location			0.101
Cervical spine	2 (8.00)	5 (11.36)	
Thoracic spine	10 (40.00)	16 (36.36)	
Lumber spine	5 (20.00)	19 (43.18)	
Cervical-thoracic spine	3 (12.00)	2 (4.55)	
Thoracic-lumber spine	5 (20.00)	2 (4.55)	
Number of involved vertebra(e)			0.376
1–2	15 (60.00)	31 (70.45)	
≥ 3	10 (40.00)	13 (29.55)	
Spinal cord compression			0.424
Yes	2 (8.00)	8 (18.18)	
No	23 (92.00)	36 (81.82)	
Pretreatment pain severity			0.154
No pain (0)	6 (24.00)	8(18.18)	
Mild pain (1–3)	10 (40.00)	13(29.55)	
Moderate pain (4–6)	5 (20.00)	8(18.18)	
Severe pain (7–10)	4 (16.00)	15(34.09)	
VCF before RT			0.106
Yes	4 (16.00)	15 (34.09)	
No	21 (84.00)	29 (65.91)	
Vertebroplasty before RT			0.755
Yes	2 (8.00)	6 (13.64)	
No	23 (92.00)	38 (86.36)	
Systemic therapy ^b^			0.252
Yes	18 (72.00)	38 (86.36)	
No	7 (28.00)	6 (13.64)	
Diphosphonate therapy			0.103
Yes	20 (80.00)	42 (95.45)	
No	5 (20.00)	2 (4.55)	
Analgesics use**^c^**			0.218
Yes	10 (52.63)	25 (69.44)	
No	9 (47.37)	11 (30.56)	

Abbreviations: ECOG = Eastern Cooperative Oncology Group; VCF = Vertebral compression fracture; RT = Radiotherapy. ^a^In the dose-escalation group, favorable type included 3 breast cancers and unfavorable type included 4 adenocarcinomas of lung, 3 neuroendocrine carcinomas, 2 nasopharyngeal carcinomas, 1 parotid adenocarcinoma, 1 osteosarcoma, 1 skin squamous cell carcinoma, 1 gastric adenocarcinoma, 1 adenocarcinoma of the duodenum, 1 hepatocellular carcinoma, 1 aggressive osteoblastoma, 1 synovial sarcoma, 1 Ewing's sarcoma, 1 alveolar soft part sarcoma, 1 malignant peripheral nerve sheath tumor, 1 malignant melanoma and 1 cancer of unknown primary site. In the 30-Gy group, the favorable type included 4 small cell lung cancers and the unfavorable type included 36 non-small cell lung cancers, 1 suprarenal epithelioma, 1 hepatocellular carcinoma, 1 adenocarcinoma of esophagus and cardia and 1 gastric adenocarcinoma. ^b^Systemic therapy refers to chemotherapy, endocrine therapy or molecular targeted therapy. ^c^Only patients with pain before radiotherapy were included in the analysis.

### Comparison of dosimetric parameters in the dose-escalation group with 30-Gy group

**Figure 1:**
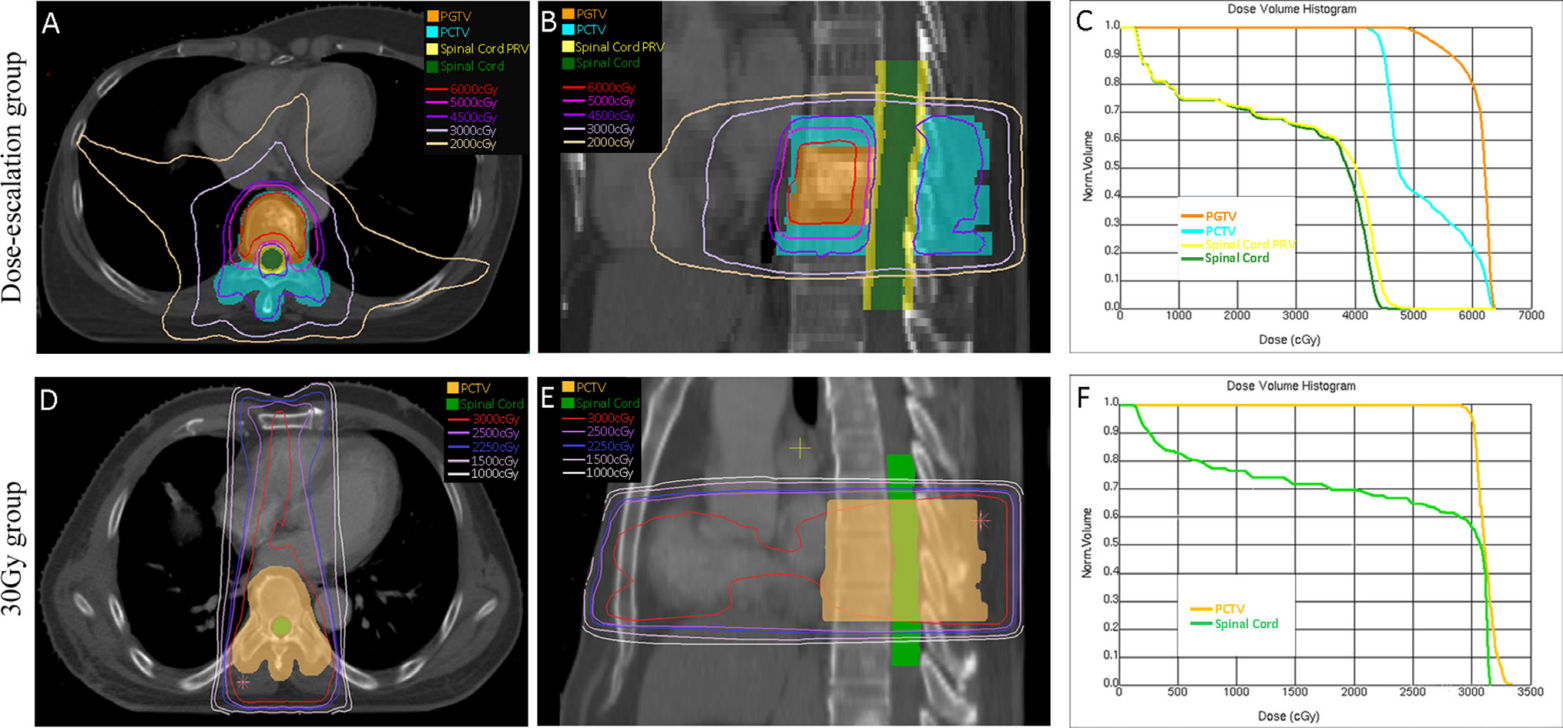
Representative target delineations, dose distributions and DVHs A metastatic lesion in the 9th thoracic vertebra was irradiated in the dose-escalation group. The prescribed dose to PGTV and PCTV were 60 Gy and 45 Gy in 23 fractions, respectively. Dose to spinal cord PRV was restricted to 48 Gy. Plan was made with IMRT in the Pinnacle V.9.2 treatment planning system and target delineation, dose distribution and DVH were shown in (**A**–**C**), respectively. A metastatic lesion in the 7th thoracic vertebra was irradiated in the 30-Gy group. The prescribed dose to PCTV was 30 Gy in 10 fractions and no restricted dose was applied to the spinal cord. One anterior field and two posterior oblique fields plan was made in the XiO Release 4.70 treatment planning system and target delineation, dose distribution and DVH were shown in (**D**–**F**), respectively.

As shown in Table 2, in the dose-escalation group, dose escalation to planning gross tumor volume (PGTV) was achieved in all patients, reaching 60 Gy for all D50s. Dmax and D2 of spinal cord planning organ at risk volume (PRV) were restricted to 48 Gy, with a maximum of 47.91 Gy and 46.56 Gy, respectively. The biologic effective doses (BEDs) of Dmax, D2 and D50 of spinal cord in the dose-escalation group were significantly lower than those in the 30-Gy group (*p* < 0.001). The homogeneity indexes (HIs) in the dose-escalation group were inferior (*p* = 0.005). Since the conformality differed significantly by using IMRT and 3D-CRT techniques, a further subgroup analysis was performed in the 30-Gy group. The conformity indexes (CIs) were superior in the dose-escalation group compared to those of the patients with 28 lesions by using 3D-CRT (*p* < 0.001) in the 30-Gy group; however, there was no significant difference in the CIs between the dose-escalation group and the 30-Gy group (with 16 lesions) by IMRT. Also, as shown in Figure 1, the target delineation, dose distribution, and dose-volume histogram (DVH) showed better results in the dose-escalation scheme compared to those of the 30-Gy regimen.

**Table 2:** Dosimetric parameters

	Dose-escalation group (*n* = 25)	30-Gy group (*n* = 44)	*P*
	Median (Range) or Mean ± SD	Median (Range) or Mean ± SD	
D2-PTV (Gy)	64.39 (61.73–69.83)	32.33 (30.27–35.96)	< 0.001
BED-Gy_10_ (Gy)	80.55 (74.43–91.99)	42.78 (37.85–48.89)	< 0.001
D50-PTV (Gy)	61.96 (60.74–67.63)	30.94 (30.04–31.93)	< 0.001
BED-Gy_10_ (Gy)	77.13 (73.04–88.42)	40.51 (36.11–42.13)	< 0.001
D95-PTV (Gy)	54.36 (41.21–60.62)	29.60 (27.39–30.14)	< 0.001
BED-Gy_10_ (Gy)	68.78 (46.87–75.71)	38.07 (34.10–39.22)	< 0.001
D98- PTV (Gy)	50.67 (35.18–59.71)	29.17 (26.94–29.77)	< 0.001
BED-Gy_10_ (Gy)	61.96 (39.31–75.21)	37.53 (33.04–38.62)	< 0.001
Dmax-Spinal cord PRV (Gy)	44.17 (31.39–47.91)^a^	—	—
BED-Gy_2_(Gy)	83.62 (47.80–98.65)^a^	—	—
D2- Spinal cord PRV (Gy)	41.41 (28.47–46.56)^a^	—	—
BED-Gy_2_ (Gy)	77.24 (41.98–93.69)^a^	—	—
D50-Spinal cord PRV (Gy)	29.38 (11.85–41.35)^a^	—	—
BED-Gy_2_ (Gy)	47.43 (14.90–78.52)^a^	—	—
Dmax- Spinal cord (Gy)	37.59 ± 4.76^a^	32.04 ± 0.80^b^	< 0.001
BED-Gy_2_ (Gy)	69.70 (36.20–88.20)^a^	83.16 (63.80–92.95)^b^	< 0.001
D2- Spinal cord (Gy)	36.11 ± 5.10 ^a^	31.83 ± 0.77^b^	0.001
BED-Gy_2_ (Gy)	64.24 (34.21–85.82)^a^	82.45 (63.59–89.82)^b^	< 0.001
D50-Spinal cord (Gy)	27.83 (11.84–39.98)^a^	30.56 (25.65–32.14)^b^	0.356
BED-Gy_2_ (Gy)	44.38 (14.89–74.73)^a^	77.25 (58.55–83.79)^b^	< 0.001
HI	0.2355 (0.0384–0.4575)	0.1095(0.0486–0.2809)	0.005
CI	0.5876 ± 0.1794 0.6393 (0.2241–0.8376)	0.2577 ± 0.1371^c^ 0.4204 (0.0831–0.8538)^d^	< 0.001 0.200

Abbreviations: SD = standard deviation; BED = biologic effective dose; PTV = planning target volume, represented PGTV in the dose-escalation group and PGTV or PCTV in the 30-Gy group; PRV = planning organ at risk volume; Dmax = maximum point dose, defined as point dose of 0.03 cc; D2 = minimal dose to 2% of the target volume; D50 = minimal dose to 50% of the target volume; D95 = minimal dose to 95% of the target volume; D98 = minimal dose to 98% of the target volume; Gy_10_ = an α/β value of 10 Gy for the tumor; Gy_2_ = an α/β value of 2 Gy for the spinal cord; HI = homogeneity index; CI = conformity index. ^a^The number of lesions was 23. Two lesions had no spinal cord segment involved in the radiation field because of the low location of the corresponding lumber vertebra(e). ^b^The number of lesions was 38. Six lesions had no spinal cord segment involved in the radiation field because of the low location of the corresponding lumber vertebra(e). ^c^28 3D-CRT plans made in PrecisePLAN Release 2.16 or XiO Release 4.70. ^d^16 IMRT plans made in Pinnacle V. 9.0 or V.9.2.

### Pain response

Only painful lesions were included for response assessment after one month of radiotherapy in the study. In addition, a patient with painful lesion in the 30-Gy group was excluded for pain assessment who underwent vertebroplasty within one month, because a vertebroplasty could also relieve pain. For one month after RT, the pain response was significantly better in the dose-escalation group than that in the 30-Gy group (*p* = 0.005, Table 3). The overall pain response rates were 94.73% and 74.29% in the dose-escalation and the 30-Gy group, respectively (*p* = 0.139). Furthermore, the complete response rate in the dose-escalation group was significantly superior to that in the 30-Gy group (73.69% vs. 34.29%, *p* = 0.006). The same result showed that the pain response was better (*p* = 0.024) and the complete response rate was higher (*p* = 0.028) in the dose-escalation group than those in the 30-Gy group, although 6 patients died of systemic progression at the three months after RT.

**Table 3:** Pain response to radiotherapy

	One month after radiotherapy			Three months after radiotherapy		
	Dose-escalation group (*n* = 19) No.(%)	30-Gy group (*n* = 35) No.(%)	*p*	Dose-escalation group (*n* = 19) No.(%)	30-Gy group (*n* = 29) No.(%)	*p*
Complete response	14 (73.69)	12 (34.29)	0.005	14 (73.69)	12 (41.38)	0.024
Partial response	4 (21.05)	14 (40.00)		4 (21.05)	11 (37.93)	
Indeterminate response	1 (5.26)	7 (20.00)		1 (5.26)	5 (17.24)	
Pain progression	0 (0.00)	2 (5.71)		0 (0.00)	1 (3.45)	

Since the Table 1 contains all patients for analysis, it does not reflect the characteristics of the pain patients. Therefore, a sub-analysis was performed for the pain patients to compare the baseline characteristics between the two groups. As a result, the findings (Supplementary Table 1) showed that there were significant differences between the two groups on the characteristics in the location of spinal metastasis and the number of involved vertebrae. In addition, to assess the risk factors that affected complete pain response, the patients were further analyzed according to the pain response. As shown in Supplementary Table 2, the result indicated that only radiation regimen was statistically different (*p* = 0.006) between two response categories. Further multivariate binary Logistic regression analysis revealed that the difference in the rates of complete pain relief was only associated with the radiotherapy regimen (*p* = 0.011, Table 4), and was independent of the other factors. In the Logistic regression analysis, the age and the location of spinal metastasis were not included in the analysis because the two factors were a minor effect on pain relief [24]. In addition, age and ECOG scores were related variables and were not simultaneously included in the multivariate analysis.

**Table 4:** Multivariate logistic regression analysis of association between clinical factors and risk of complete response

Clinical factors	Odds ratio	95% CI	*p*
Radiation regimen			
Dose-escalation vs. 30-Gy	11.145	1.722–72.121	0.011^a^
Gender			
Male vs. Female	0.401	0.078–2.055	0.273
ECOG performance status			
0 vs. 1–2	2.878	0.090–92.556	0.551
0 vs. 3–4	3.346	0.186–60.086	0.412
Primary tumor			
Unfavorable vs. Favorable	0.494	0.031–7.866	0.618
Number of involved vertebra(e)			
1–2 vs. ≥ 3	0.588	0.124–2.793	0.505
Spinal cord compression			
Yes vs. No	2.538	0.399–16.133	0.324
Pretreatment pain severity			
Mild vs. Moderate	0.063	0.030–1.391	0.080
Mild vs. Severe	0.362	0.054–2.452	0.298
VCF before RT			
Yes vs. No	0.608	0.125–2.967	0.539
Vertebroplasty before RT			
Yes vs. No	0.587	0.059–5.798	0.649
Systemic therapy^b^			
Yes vs. No	0.927	0.138–6.220	0.938
Diphosphonate therapy			
Yes vs. No	15.811	0.863–289.654	0.063
Analgesics use			
Yes vs. No	4.892	0.290–82.415	0.271

Abbreviations: CI = confidence interval; ECOG = Eastern Cooperative Oncology Group; VCF = Vertebral compression fracture; RT = Radiotherapy; ^a^*p*-value was less than 0.05 and difference was considered statistically significant. ^b^Systemic therapy refers to chemotherapy, endocrine therapy or molecular targeted therapy.

### Duration of pain relief

All lesions with complete or partial response in Table 3 were included for assessing the duration of pain relief. One patient with partial response in the 30-Gy group was excluded because the vertebroplasty was operated for further pain control. There was no statistical difference in follow-up period between the two groups. The median duration of pain relief was 258 days (range 60–835) in the dose-escalation group, longer than the 171 days (range 8–773) in the 30-Gy group, although without statistical difference (*p* = 0.555). Pain relapse were 16.67% (3/18) and 20.00% (5/25) in the dose-escalation and the 30-Gy group, respectively.

### Acute and late complications

Acute radiation toxicities occurred in 12.00% (3/25) and 20.45% (9/44) lesions in the dose-escalation and the 30-Gy group, respectively (*p* = 0.575). One Grade 1 esophageal reaction, one Grade 1 upper gastrointestinal reaction, and one Grade 1 skin reaction occurred in the dose-escalation group. In the 30-Gy group, three Grade 1 skin reactions, two Grade 1 esophageal reactions, one Grade 1 laryngeal reaction, one Grade 1 upper gastrointestinal reaction, one Grade 2 upper gastrointestinal reaction, and one Grade 4 oral mucosal reaction were observed.

No late radiation complications such as radiation-induced myelopathy were observed in both groups.

### Overall survival

The follow-up periods were of no statistical difference between the two groups (*p* = 0.054), although the median in the dose-escalation group was 376 days (range 90–898), a bit longer than the 219 days (range 28–881) in the 30-Gy group. The overall follow-up rate was 94.2% (65/69). In the dose-escalation group, 24 lesions completed the follow-ups, among which 8 were still alive at the last follow-up and 16 died of systemic progression, whereas 1 lost the follow-ups 140 days after radiotherapy. In the 30-Gy group, 41 lesions completed the follow-ups, among which 10 were still alive at the last follow-up and 31 died of systemic progression, whereas 3 lost the follow-ups after 244, 177, 87 days of radiotherapy, respectively.

The median overall survivals (OSs) were 418 days in the dose-escalation and 324 days in the 30-Gy group, with a trend of better survival in the dose-escalation group, though without statistical difference (*p* = 0.053, Supplementary Figure 1).

## DISCUSSION

Reports on the feasibility of dose escalation using conventionally-fractionated IG-IMRT in spinal metastases have already been taken out [23, 25]. However, relevant clinical outcomes have rarely been studied [26]. Previous studies were single-arm studies with a small volume of samples. Here, it is the first report that the dosimetric parameters and clinical outcomes are compared between the dose-escalation regimen and the commonly used 30-Gy protocol. Furthermore, this study has a large sample size in the dose-escalation group and all the patients were treated above 60 Gy. The results for the study have demonstrated that the dose-escalation regimen was superior to the 30-Gy protocol in dosimetric parameters and pain response, especially the complete pain response rate.

The dose-escalation regimen indicated obvious advantages in dosimetric parameters. It achieved dose escalation to PGTV with all D50s beyond 60 Gy, which was consistent with our previous results [23]. As a result, the higher the radiation dose was administered to the metastatic sites, the better the pain response and the tumor control were. In terms of the spinal cord, each Dmax and D2 of spinal cord PRV met prescription requirement in the dose-escalation group. This study demonstrated for the first time that the corresponding BEDs of Dmax, D2 and D50 of the spinal cord in the dose-escalation group were significantly less than those in the 30-Gy group. The results seemed questionable because the Dmax and D2 of the spinal cord were higher in the dose-escalation group. However, when corresponding BEDs were calculated using the formula BED = D × [1+d/(α/β)], the twice fractions in the dose escalation group can counteract the slightly higher dose of spinal cord. More importantly, BEDs of Dmax of the spinal cord in the dose-escalation group were all less than the safe BED of 98 Gy for spinal cord in each treatment course in re-irradiation [27]. This suggests that the risk of spinal cord injury will be smaller in the dose-escalation group when re-irradiation is needed. The superior CI in the dose-escalation group is the basis for dosimetric superiority.

Pain response, the main clinical outcome, was also significantly better in the dose-escalation group. In this study, the criteria of pain response established by the International Bone Metastases Consensus Working Party [28, 29] was adopted. Changes both in numerical rating scale (NRS) and analgesics dosing were taken into account, making it easier to compare the results with those of other studies using the consensus as well. Patients with mild pain were included into the pain response analysis according to the consensus because their NRS were all 2 to 3 [28]. Although the updated consensus suggested that a minimum score of 5 may be a better criterion, the new suggestion was still opposed by 31% experts for fear that it would restrict entry of patients with higher pain tolerance [29]. The overall response rate in the dose-escalation group was higher than that in the conventional radiotherapy [8, 16]. More importantly, the complete response rate increased significantly. This suggests that dose-escalation regimen would benefit more patients with complete response rather than partial response.

The median duration of pain relief was 171 days in the 30-Gy group, consistent with the result of another study [30]. The median duration of pain relief was 258 days in the dose-escalation group in our study, which was longer than that in the 30-Gy group, although this was not statistically significant. However, the results need to be interpreted with precautions. Few lesions relapsed in both groups due to patients’ death and short follow-up, which could hardly be avoided among patients with distant metastases. Also, longer observation time in the dose-escalation group could also lead to the longer duration of pain relief. Further studies in patients with longer life expectancy are needed.

No radiation-induced myelopathy was observed in the dose-escalation group, confirming the dosimetric results that all spinal cords were within tolerance. It also proved the advantage of IGRT in dose-escalation regimen [23, 25], because doses decrease so sharply in IMRT that even set-up errors less than 3 mm would cause huge dose changes in spinal cord [25]. Radiation-induced myelopathy was also not observed during a median follow-up of 15.6 or 17 months in previous dose-escalation studies [23, 26]. However, the incidence of radiation-induced myelopathy might be underreported because of short survival of these patients. The acute radiation complications were mild and skin or gastrointestinal reaction remained the most common, which was consistent with other studies [30, 31]. Taken together, toxicities in the dose-escalation group were not higher than those in the 30-Gy group, indicating that dose escalation beyond 60 Gy with IG-IMRT is safe.

OS was determined mainly by the nature of the primary tumor and the effectiveness of systemic treatment. Therefore, it is not surprising that the OS was of no statistical difference between the two groups. It demonstrated the fact that it remains difficult to develop an effective predictive model for life expectancy in patients with spinal metastases [32–34]. An effective predictive model for life expectancy will be helpful to guide the radiation regimen selection for spinal metastases in these patients.

Stereotactic body radiation therapy (SBRT) could result in similar clinical outcomes as dose-escalation regimen. The overall pain response rate is about 80% [35, 36] and the local control rate is 90%–95% [35–37]. However, the incidence of vertebral compression fracture (VCF) could be as high as 11%–39% [38–40]. It is much higher than the incidence of 5% in conventional radiotherapy [8]. VCF occurs at 3 to 25 months after SBRT [38–40], but no VCF was observed during the 1-year and 2-year follow-up period in this study and in Guckenberger's dose-escalation study, respectively [26]. The absence of VCF might be the advantage of dose-escalation radiotherapy over SBRT.

Several limitations should be considered when interpreting the results. First, the histological types of primary tumors were diverse in the dose-escalation group while most patients in the 30-Gy group were with non-small cell lung cancer. Both groups lacked common types such as breast cancer and prostate cancer [6]. Second, there existed confounding factors such as more severe pain in the 30-Gy group and inconformity of analgesics application between two groups, although they did not affect complete pain response with the Logistic regression analysis. Third, data on local tumor control rate and health economy were not collected, which were difficult to obtain in a retrospective study and will be the focus of future studies. Prospective multicenter study with a larger patient population and a longer life expectancy should be conducted in the future.

In conclusion, in comparison with the 30-Gy regimen, the dose-escalation regimen with conventionally-fractionated IG-IMRT showed a trend of achieving better dosimetric parameters, increasing pain relief and potentially improving the quality of life for patients with spinal metastases, and thus being recommended for these patients, especially those with a long life expectancy.

## MATERIALS AND METHODS

### Patients and treatment

Sixty-nine metastatic spinal lesions treated with radiotherapy at the West China Hospital from March 1, 2010 to July 17, 2014 were enrolled, among which 25 were in the dose-escalation group and 44 were in the 30-Gy group. This study was approved by the Research Ethics Committee of West China Hospital. Primary tumors were classified into favorable and unfavorable tumors according to literature [12]. Favorable histologic primary tumors included breast and prostate carcinoma; small cell lung cancer; lymphoma and myeloma. Unfavorable histologic primary tumors included non-small cell lung, gastric, colon-rectal, liver, head and neck, kidney, bladder and uterine carcinoma; melanoma; sarcoma; and others.

IG-IMRT was performed in all patients in the dose-escalation group. The prescribed doses to PGTV were ≥ 60 Gy. While for the 30-Gy group as a control, patients received 30 Gy in 10 fractions with the three-dimensional conformal radiotherapy (3D-CRT) or 3–5 field IMRT.

The radiation administration, treatment planning, and image guidance in dose-escalation group were described previously [23]. Briefly, gross tumor volume (GTV) included the metastatic lesions, and the half vertebra above and below the metastatic vertebra (e) were covered as clinical target volume (CTV). A 3mm margin was expanded isotropically from GTV or CTV to form PGTV or planning clinical target volume (PCTV). A dose of 60–66 Gy was given to PGTV in 20 to 30 fractions, and the dose to spinal cord PRV was restricted to 45–48 Gy.

### Follow-up and data collection

Dmax, D2, D50, D95, and D98 were extracted from tabular DVH. HI [41], CI [42], and BED [26] were calculated. Biologic effective dose (BED) = D × [1+d/(α/β)], whereas D = total dose and d = dose per fraction. α/β values of 10 Gy and 2 Gy were hypothesized for the tumor and the spinal cord, respectively. Follow-ups were carried out at 1 month, 3 months and thereafter every 3 months after radiotherapy by clinical visits or phone calls until death or January 31, 2015 among survivors, ensuring a minimum follow-up of 6 months for each survivor. Pain was assessed by the worst pain without administration of analgesics before radiotherapy evaluated by the 11-point NRS and the 4-point categorical verbal rating scale (VRS) [43]. Pain response was first evaluated 30 days after radiotherapy in alignment with the consensus released by the International Bone Metastases Consensus Working Party [28, 29]. Complete response, partial response, indeterminate response and pain progression were defined as reported in the consensus [28, 29]. Overall response included complete and partial response. Pain relapse was defined as any occurrence of pain in patients with complete response and pain progression in those with partial response. Duration of pain relief was evaluated in patients with complete and partial response, and was calculated from the first date evaluated at 30 days after radiotherapy to the date of relapse, or the date of death, or last follow-up. OS was calculated from the date of completion of radiotherapy.

Acute and late radiation complications were assessed according to Radiation Therapy Oncology Group (RTOG) Acute Radiation Morbidity Scoring Criteria and RTOG/ European Organization for Research on Treatment of Cancer (EORTC) Late Radiation Morbidity Scoring Schema [44].

### Statistical analysis

IBM SPSS statistics for Windows version 19.0 (IBM Corp., Armonk, NY, USA) was applied for statistical analysis. Mann-Whitney *U* test or Student's *t* test was used for comparing the quantitative data. Chi-square test and Wilcoxon rank sum test were applied to compare the qualitative and ranked data, respectively. Kaplan-Meier method with a log-rank test was used to assess the survival. Logistic regression was used to analyze the risk factors that might affect the pain relief. A two-sided *p*-value of < 0.05 was considered as statistically significant.

## SUPPLEMENTARY MATERIALS FIGURES AND TABLES



## Abbreviations

3D-CRT: three-dimensional conformal radiotherapy
BED: biological effective dose
CBCT: cone beam computer tomography
cc: cubic centimeter
CI: conformity index
CI: confidence interval
CTV: clinical target volume
Dmax: maximum point dose
D2: minimal dose to 2% of the target volume
D50: minimal dose to 50% of the target volume
D95: minimal dose to 95% of the target volume
D98: minimal dose to 98% of the target volume
DVH: dose-volume histogram
ECOG: Eastern Cooperative Oncology Group
EORTC: European Organization for Research on Treatment of Cancer
f: fraction
GTV: gross tumor volume
HI: homogeneity index
IGRT: image-guided radiotherapy
IG-IMRT: image-guided intensity-modulated radiotherapy
IMRT: intensity-modulated radiation therapy
NRS: numerical rating scale
PCTV: planning clinical target volume
PGTV: planning gross tumor volume
PRV: planning organ at risk volume
RT: radiotherapy
RTOG: Radiation Therapy Oncology Group
SBRT: stereotactic body radiation therapy
SD: standard deviation
OS: overall survival
VCF: vertebral compression fracture
VRS: verbal rating scale

## References

[1] Witham TF, Khavkin YA, Gallia GL, Wolinsky JP, Gokaslan ZL (2006). Surgery insight: current management of epidural spinal cord compression from metastatic spine disease. Nat Clin Pract Neurol.

[2] Hatrick NC, Lucas JD, Timothy AR, Smith MA (2000). The surgical treatment of metastatic disease of the spine. Radiother Oncol.

[3] Ortiz Gomez JA (1995). The incidence of vertebral body metastases. Int Orthop.

[4] Helweg-Larsen S, Sorensen PS (1994). Symptoms and signs in metastatic spinal cord compression: a study of progression from first symptom until diagnosis in 153 patients. Eur J Cancer.

[5] Jacobs WB, Perrin RG (2001). Evaluation and treatment of spinal metastases: an overview. Neurosurg Focus.

[6] Bhatt AD, Schuler JC, Boakye M, Woo SY (2013). Current and emerging concepts in non-invasive and minimally invasive management of spine metastasis. Cancer Treat Rev.

[7] Simmons ED, Zheng Y (2006). Vertebral tumors: surgical versus nonsurgical treatment. Clin Orthop Relat Res.

[8] Chow E, Harris K, Fan G, Tsao M, Sze WM (2007). Palliative radiotherapy trials for bone metastases: a systematic review. J Clin Oncol.

[9] Chow E, Zeng L, Salvo N, Dennis K, Tsao M, Lutz S (2012). Update on the systematic review of palliative radiotherapy trials for bone metastases. Clin Oncol (R Coll Radiol).

[10] Rades D, Stalpers LJ, Veninga T, Schulte R, Hoskin PJ, Obralic N, Bajrovic A, Rudat V, Schwarz R, Hulshof MC, Poortmans P, Schild SE (2005). Evaluation of five radiation schedules and prognostic factors for metastatic spinal cord compression. J Clin Oncol.

[11] Rades D, Fehlauer F, Stalpers LJ, Wildfang I, Zschenker O, Schild SE, Schmoll HJ, Karstens JH, Alberti W (2004). A prospective evaluation of two radiotherapy schedules with 10 versus 20 fractions for the treatment of metastatic spinal cord compression: final results of a multicenter study. Cancer.

[12] Maranzano E, Bellavita R, Rossi R, De Angelis V, Frattegiani A, Bagnoli R, Mignogna M, Beneventi S, Lupattelli M, Ponticelli P, Biti GP, Latini P (2005). Short-course versus split-course radiotherapy in metastatic spinal cord compression: results of a phase III, randomized, multicenter trial. J Clin Oncol.

[13] Maranzano E, Trippa F, Casale M, Costantini S, Lupattelli M, Bellavita R, Marafioti L, Pergolizzi S, Santacaterina A, Mignogna M, Silvano G, Fusco V (2009). 8Gy single-dose radiotherapy is effective in metastatic spinal cord compression: results of a phase III randomized multicentre Italian trial. Radiother Oncol.

[14] Popovic M, den Hartogh M, Zhang L, Poon M, Lam H, Bedard G, Pulenzas N, Lechner B, Chow E (2014). Review of international patterns of practice for the treatment of painful bone metastases with palliative radiotherapy from 1993 to 2013. Radiother Oncol.

[15] Rades D, Karstens JH, Hoskin PJ, Rudat V, Veninga T, Schild SE, Dunst J (2007). Escalation of radiation dose beyond 30 Gy in 10 fractions for metastatic spinal cord compression. Int J Radiat Oncol Biol Phys.

[16] Wu JS, Wong R, Johnston M, Bezjak A, Whelan T (2003). Meta-analysis of dose-fractionation radiotherapy trials for the palliation of painful bone metastases. Int J Radiat Oncol Biol Phys.

[17] Hayat MJ, Howlader N, Reichman ME, Edwards BK (2007). Cancer statistics, trends, and multiple primary cancer analyses from the Surveillance, Epidemiology, and End Results (SEER) Program. Oncologist.

[18] 8 Gy single fraction radiotherapy for the treatment of metastatic skeletal pain: randomised comparison with a multifraction schedule over 12 months of patient follow-up. Bone Pain Trial Working Party (1999). Radiother Oncol.

[19] Rades D, Lange M, Veninga T, Stalpers LJ, Bajrovic A, Adamietz IA, Rudat V, Schild SE (2011). Final results of a prospective study comparing the local control of short-course and long-course radiotherapy for metastatic spinal cord compression. Int J Radiat Oncol Biol Phys.

[20] Schultheiss TE, Kun LE, Ang KK, Stephens LC (1995). Radiation response of the central nervous system. Int J Radiat Oncol Biol Phys.

[21] Nutting C, Dearnaley DP, Webb S (2000). Intensity modulated radiation therapy: a clinical review. Br J Radiol.

[22] Thilmann C, Nill S, Tucking T, Hoss A, Hesse B, Dietrich L, Bendl R, Rhein B, Haring P, Thieke C, Oelfke U, Debus J, Huber P (2006). Correction of patient positioning errors based on in-line cone beam CTs: clinical implementation and first experiences. Radiat Oncol.

[23] Gong Y, Wang J, Bai S, Jiang X, Xu F (2008). Conventionally-fractionated image-guided intensity modulated radiotherapy (IG-IMRT): a safe and effective treatment for cancer spinal metastasis. Radiat Oncol.

[24] Westhoff PG, de Graeff A, Reyners AK, Monninkhof EM, Rodenhuis CC, van Vulpen M, Leer JW, Marijnen CA, van der Linden YM (2014). Effect of age on response to palliative radiotherapy and quality of life in patients with painful bone metastases. Radiother Oncol.

[25] Guckenberger M, Meyer J, Wilbert J, Baier K, Bratengeier K, Vordermark D, Flentje M (2007). Precision required for dose-escalated treatment of spinal metastases and implications for image-guided radiation therapy (IGRT). Radiother Oncol.

[26] Guckenberger M, Goebel J, Wilbert J, Baier K, Richter A, Sweeney RA, Bratengeier K, Flentje M (2009). Clinical outcome of dose-escalated image-guided radiotherapy for spinal metastases. Int J Radiat Oncol Biol Phys.

[27] Nieder C, Grosu AL, Andratschke NH, Molls M (2006). Update of human spinal cord reirradiation tolerance based on additional data from 38 patients. Int J Radiat Oncol Biol Phys.

[28] Chow E, Wu JS, Hoskin P, Coia LR, Bentzen SM, Blitzer PH (2002). International consensus on palliative radiotherapy endpoints for future clinical trials in bone metastases. Radiother Oncol.

[29] Chow E, Hoskin P, Mitera G, Zeng L, Lutz S, Roos D, Hahn C, van der Linden Y, Hartsell W, Kumar E (2012). Update of the international consensus on palliative radiotherapy endpoints for future clinical trials in bone metastases. Int J Radiat Oncol Biol Phys.

[30] Foro Arnalot P, Fontanals AV, Galceran JC, Lynd F, Latiesas XS, de Dios NR, Castillejo AR, Bassols ML, Galan JL, Conejo IM, Lopez MA (2008). Randomized clinical trial with two palliative radiotherapy regimens in painful bone metastases: 30 Gy in 10 fractions compared with 8 Gy in single fraction. Radiother Oncol.

[31] Hartsell WF, Scott CB, Bruner DW, Scarantino CW, Ivker RA, Roach M, Suh JH, Demas WF, Movsas B, Petersen IA, Konski AA, Cleeland CS, Janjan NA, DeSilvio M (2005). Randomized trial of short- versus long-course radiotherapy for palliation of painful bone metastases. J Natl Cancer Inst.

[32] Rades D, Fehlauer F, Schulte R, Veninga T, Stalpers LJ, Basic H, Bajrovic A, Hoskin PJ, Tribius S, Wildfang I, Rudat V, Engenhart-Cabilic R, Karstens JH (2006). Prognostic factors for local control and survival after radiotherapy of metastatic spinal cord compression. J Clin Oncol.

[33] Rades D, Douglas S, Veninga T, Stalpers LJ, Hoskin PJ, Bajrovic A, Adamietz IA, Basic H, Dunst J, Schild SE (2010). Validation and simplification of a score predicting survival in patients irradiated for metastatic spinal cord compression. Cancer.

[34] Aoude A, Amiot LP (2014). A comparison of the modified Tokuhashi and Tomita scores in determining prognosis for patients afflicted with spinal metastasis. Can J Surg.

[35] Gerszten PC, Burton SA, Ozhasoglu C, Welch WC (1976). Radiosurgery for spinal metastases: clinical experience in 500 cases from a single institution. Spine (Phila Pa.

[36] Hall WA, Stapleford LJ, Hadjipanayis CG, Curran WJ, Crocker I, Shu HK (2011). Stereotactic body radiosurgery for spinal metastatic disease: an evidence-based review. Int J Surg Oncol.

[37] Amdur RJ, Bennett J, Olivier K, Wallace A, Morris CG, Liu C, Mendenhall WM (2009). A prospective, phase II study demonstrating the potential value and limitation of radiosurgery for spine metastases. Am J Clin Oncol.

[38] Cunha MV, Al-Omair A, Atenafu EG, Masucci GL, Letourneau D, Korol R, Yu E, Howard P, Lochray F, da Costa LB, Fehlings MG, Sahgal A (2012). Vertebral compression fracture (VCF) after spine stereotactic body radiation therapy (SBRT): analysis of predictive factors. Int J Radiat Oncol Biol Phys.

[39] Rose PS, Laufer I, Boland PJ, Hanover A, Bilsky MH, Yamada J, Lis E (2009). Risk of fracture after single fraction image-guided intensity-modulated radiation therapy to spinal metastases. J Clin Oncol.

[40] Boehling NS, Grosshans DR, Allen PK, McAleer MF, Burton AW, Azeem S, Rhines LD, Chang EL (2012). Vertebral compression fracture risk after stereotactic body radiotherapy for spinal metastases. J Neurosurg Spine.

[41] Xiao J, Li Y, Jiang Q, Sun L, Henderson F, Wang Y, Jiang X, Li G, Chen N (2013). Hepatic arterial phase and portal venous phase computed tomography for dose calculation of stereotactic body radiation therapy plans in liver cancer: a dosimetric comparison study. Radiat Oncol.

[42] Lee TF, Chao PJ, Fang FM, Su TJ, Leung SW, Hsu HC (2010). Helical tomotherapy for single and multiple liver tumours. Radiat Oncol.

[43] Breivik H, Borchgrevink PC, Allen SM, Rosseland LA, Romundstad L, Hals EK, Kvarstein G, Stubhaug A (2008). Assessment of pain. Br J Anaesth.

[44] Cox JD, Stetz J, Pajak TF (1995). Toxicity criteria of the Radiation Therapy Oncology Group (RTOG) and the European Organization for Research and Treatment of Cancer (EORTC). Int J Radiat Oncol Biol Phys.

