# Supplementary material for: Dose escalation by image-guided intensity-modulated radiotherapy leads to an increase in pain relief for spinal metastases: a comparison study with a regimen of 30 Gy in 10 fractions
Source: Oncotarget. 2017 Jul 4;8(68):112330–40. doi: 10.18632/oncotarget.18979 (PMC5762513; doi:10.18632/oncotarget.18979)
Supplement: Supplementary file 1 [file oncotarget-08-112330-s001.pdf]

Dose escalation by image-guided intensity-modulated radiotherapy leads to an increase in pain relief for spinal metastases: a comparison study with a regimen of 30 Gy in 10 fractions

SUPPLEMENTARY MATERIALS

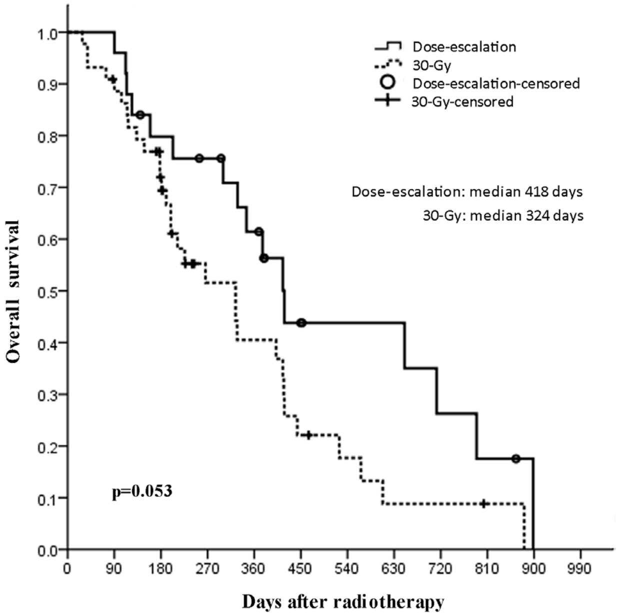

Number of patients at risk

Dose-escalation	24	19	17	13	6	5	5	3	2	0
30-Gy	38	28	14	11	6	4	2	2	2	0

Supplementary Figure 1: Overall survival.

Supplementary Table 1: Characteristics in patients included in pain response analysis between two radiotherapy groups

	Dose-escalation group (n = 19) No. of lesions (%)	30-Gy group (n = 35) No. of lesions (%)	p
Age (years)			0.354
< 60	16 (68.42)	24 (68.57)	
≥ 60	3 (31.58)	11 (31.43)	
Gender			0.313
Male	13 (68.42)	19 (54.29)	
Female	6 (31.58)	16 (45.71)	
ECOG performance status			0.230
0	6 (31.58)	6 (17.14)	
1–2	12 (63.16)	26 (74.29)	
3–4	1 (5.26)	3 (8.57)	
Primary tumor			> 0.999
Unfavorable	17 (89.47)	32 (90.91)	
Favorable	2 (10.53)	3 (9.09)	
Spinal metastasis location			0.028[a]
Cervical spine	1 (5.260)	5 (11.36)	
Thoracic spine	6 (31.58)	10 (36.36)	
Lumber spine	4 (21.05)	17 (43.18)	
Cervical-Thoracic spine	3 (15.79)	2 (4.55)	
Thoracic-Lumber spine	5 (26.32)	1 (4.55)	
Number of involved vertebra(e)			0.002[a]
1–2	9 (47.37)	25 (71.43)	
≥ 3	10 (52.63)	10 (28.57)	
Spinal cord compression			0.610
Yes	2 (10.53)	7 (20.00)	
No	17 (89.47)	28 (80.00)	
Pretreatment pain severity			0.145
Mild pain (1-3)	10 (52.63)	13 (37.14)	
Moderate pain (4-6)	5 (26.32)	7 (20.00)	
Severe pain (7-10)	4 (21.05)	15 (42.86)	
VCF before RT			0.224
Yes	4 (21.05)	13 (37.14)	
No	15 (78.95)	22 (62.86)	
Vertebroplasty before RT			0.801
Yes	2 (10.53)	6 (17.14)	
No	17 (89.47)	29 (82.86)	
Systemic therapy [b]			0.381
Yes	13 (68.42)	29 (82.86)	
No	6 (31.58)	6 (17.14)	
Diphosphonate therapy			0.087
Yes	15 (78.95)	34 (97.14)	
No	4 (21.05)	1 (2.86)	
Analgesics use			0.247
Yes	10 (52.63)	24 (68.57)	
No	9 (47.37)	11 (31.43)	
Daily oral morphine equivalent (mg) [c]			0.624
Median (Range)	80 (60–120)	80 (40–180)	

Abbreviations: ECOG = Eastern Cooperative Oncology Group; VCF= Vertebral compression fracture; RT = Radiotherapy.
[a] p-value was less than 0.05 and difference was considered statistically significant.
[b] Systemic therapy refers to chemotherapy, endocrine therapy or molecular targeted therapy.
[c] All narcotic analgesics were converted to daily oral morphine equivalent for analgesic scoring.

Supplementary Table 2: Reorganization of characteristics in patients included in pain response analysis by complete response

	Complete response (n = 26) No. of lesions (%)	Others[a] (n = 28) No. of lesions (%)	p
Radiation regimen			0.006[b]
Dose-escalation	14 (53.85)	5 (17.86)	
30-Gy	12 (46.15)	23 (82.14)	
Age (years)			0.089
< 60	22 (84.62)	18 (64.29)	
≥ 60	4 (15.38)	10 (35.71)	
Gender			0.435
Male	14 (53.85)	18 (64.29)	
Female	12 (46.15)	10 (35.71)	
ECOG performance status			0.243
0	8 (30.77)	4 (14.29)	
1–2	16 (61.54)	22 (78.57)	
3–4	2 (7.69)	2 (7.14)	
Primary tumor			0.931
Unfavorable	23 (88.46)	26 (92.86)	
Favorable	3 (11.54)	2 (7.14)	
Spinal metastasis location			0.137
Cervical spine	3 (11.54)	3 (10.71)	
Thoracic spine	5 (19.23)	11 (39.29)	
Lumber spine	9 (34.62)	12 (42.86)	
Cervical-Thoracic spine	4 (15.38)	1 (3.57)	
Thoracic-Lumber spine	5 (19.23)	1 (3.57)	
Number of involved vertebra(e)			0.181
1–2	14 (53.85)	20 (71.43)	
≥ 3	12 (46.15)	8 (28.57)	
Spinal cord compression			0.903
Yes	5 (19.23)	4 (14.29)	
No	21 (80.77)	24 (85.71)	
Pretreatment pain severity			0.063
Mild pain (1–3)	14 (53.85)	9 (32.14)	
Moderate pain (4–6)	6 (23.08)	6 (21.43)	
Severe pain (7–10)	6 (23.07)	13 (46.43)	
VCF before RT			0.487
Yes	7 (26.92)	10 (35.71)	
No	19 (73.08)	18 (64.29)	
Vertebroplasty before RT			0.999
Yes	4 (15.38)	4 (14.29)	
No	22 (84.62)	24 (85.71)	
Systemic therapy[c]			0.610
Yes	21 (80.77)	21 (75.00)	
No	5 (19.23)	7 (25.00)	
Diphosphonate therapy			0.999
Yes	24 (92.31)	25 (89.29)	
No	2 (7.69)	3 (10.71)	
Analgesics use			0.440
Yes	15 (57.69)	19 (67.86)	
No	11 (42.31)	9 (32.14)	

Abbreviations: ECOG=Eastern Cooperative Oncology Group; VCF= Vertebral compression fracture; RT=Radiotherapy.
[a]Others included partial response, indeterminate response and pain progression.
[b]p-value was less than 0.05 and difference was considered statistically significant.
[c]Systemic therapy refers to chemotherapy, endocrine therapy or molecular targeted therapy.
